# Monolithically Integrated Perovskites for High-Performance Vapor-Deposited Narrowband Photodetectors

**DOI:** 10.1021/acsphotonics.6c01728

**Published:** 2026-09-28

**Authors:** Serhii Derenko, Lidón Gil-Escrig, Joost W. C. Reinders, Cristina Roldán-Carmona, Daniel Tordera, Michele Sessolo

**Affiliations:** Instituto de Ciencia Molecular, 16781 Universidad de Valencia, C/Catedrático J. Beltrán 2, 46980 Paterna, Spain

**Keywords:** perovskite, photodiode, photodetector, narrowband, tandem, monolithic integration, vapor deposition, optical filter

## Abstract

Narrowband photodetectors, owing to their intrinsic spectral selectivity, are essential for applications such as optical communication and imaging. One strategy to preserve the high quantum efficiency of thin photodiodes is to combine them with optical filters. However, externally integrated filters increase device complexity and compromise robustness, while electrically active integrated filters generate parasitic photocurrent that limits spectral rejection. Here, we monolithically integrate an optically active but electrically passive perovskite filter within the photodetector architecture, and compare its performance with that of a conventional external filter configuration. In-depth characterization of both device designs shows that the integrated architecture preserves the performance of the active photodiode while providing narrowband operation, with a full width at half maximum (FWHM) of 50 nm at 750 nm and a specific detectivity of 1 × 10^13^ Jones at −0.5 V. Beyond its higher responsivity, the integrated device shows no observable filter degradation after three months of storage in air, in contrast to the nonintegrated configuration. These results demonstrate that monolithic integration of an electrically passive perovskite filter enables stable, high-performance narrowband photodetectors without compromising device performance.

## Graphical abstract

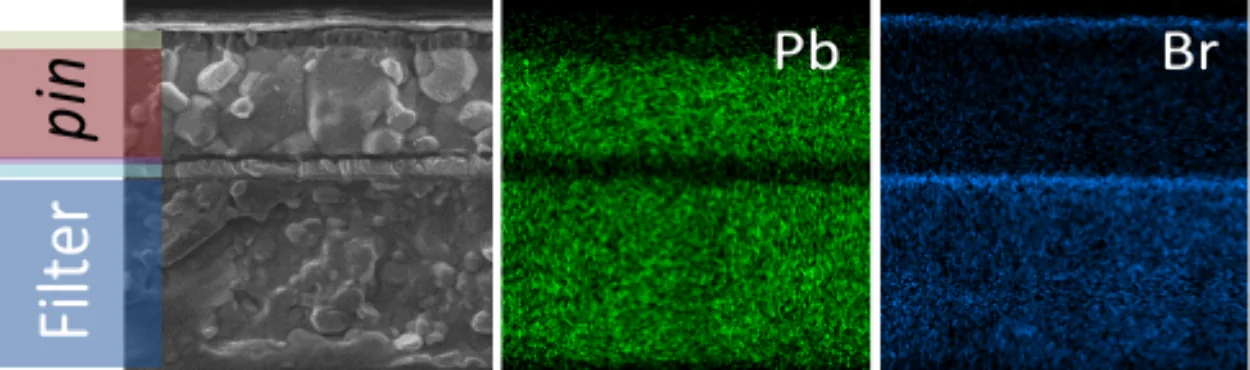

## Introduction

Photodetectors (PDs) are devices that convert photons into measurable electrical signals.^1^ Perovskite-based photodetectors have already demonstrated performance comparable to established technologies such as silicon-based detectors, making them attractive candidates for next-generation optoelectronic applications.^2^ Metal-halide perovskites (MHPs), a class of semiconductors with the general formula ABX_3_ (where A is a small organic cation or Cs^+^, B is a divalent metal such as Pb^2+^ or Sn^2+^, and X is a halide), are promising candidates as active materials in PDs due to their high absorption coefficients,^3^ tunable bandgap,^4,5^ long carrier diffusion lengths,^6,7^ and defect tolerance,^8,9^ which enable efficient charge collection. Their optical properties can be readily tuned by modifying their composition, particularly the halide content, enabling spectral response from the visible to the near-infrared. Although MHP thin films can be deposited at low temperatures using solution-based methods, these approaches remain challenging to scale while maintaining film uniformity and reproducibility, and the use of solvents intrinsically complicates the fabrication of multilayer device architectures. In contrast, vapor deposition enables low-temperature growth of high-quality perovskite films on virtually any substrate while providing excellent control over film thickness and composition and greatly simplifying the fabrication of complex multilayer devices.^10–13^

Among the many applications of photodetectors, some require spectrally selective detection rather than a broadband response. Examples include digital cameras, multispectral machine vision, artificial retinas, and biomedical fluorescence imaging, where closely spaced wavelengths must be distinguished to reconstruct an image or isolate a target signal from the background.^14–16^ In optical communication, including free-space visible-light communication (VLC), Li-Fi, and short-haul wavelength-division multiplexing, narrowband detectors enable independent read-out of channels encoded on different wavelengths and reduce ambient-light interference, which directly improves the signal-to-noise ratio and therefore data rate.^12^ Conventional image sensors achieve this by placing a Bayer color-filter array or an external bandpass filter in front of a broadband silicon photodiode, at the cost of optical losses, pixel-level alignment, and additional processing steps that limit further miniaturization.^17^

Several strategies have been reported to realize narrowband photoresponse in perovskite photodetectors. Narrowband detection can be obtained by exploiting the thickness of a broadband absorber through the charge-collection-narrowing (CCN) mechanism, in which short-wavelength photons are absorbed close to the front electrode and lost to surface recombination, while only near-bandgap photons generate collectable carriers.^18,19^ CCN in solution-processed perovskites has delivered tunable red, green, and blue response with FWHMs below ∼100 nm, but the required junction thicknesses (from tens of micrometres up to ∼1 mm for single-crystal variants) produce slow response times and low external quantum efficiency (EQE), often only a few percent, because carriers generated far from the collecting contacts cannot be efficiently extracted. Another strategy is to enhance a narrow spectral region through microcavity or plasmonic resonance effects,^20,21^ which can deliver very sharp response peaks and sub-wavelength footprints but rely on (nano-)­fabrication steps (metallic nanostructures, distributed Bragg reflectors) that complicate scale-up and tie the spectral position to the resonator geometry rather than to the absorber itself. Alternatively, a broadband photodetector can be coupled with an external optical filter,^22^ so that the effective spectral response corresponds to the overlap between the EQE spectrum and the filter transmission. Such devices are simple to conceive but typically suffer from reduced responsivity due to filter losses and require additional alignment, bonding or encapsulation steps that are incompatible with compact, large-area arrays. More recently, the filter layer has been directly integrated into the device stack in the form of a photoconductor.^23^ This architecture demonstrated the feasibility of monolithic integration, but because the filter also participates in charge generation and transport, absorbed photons within the filter contribute to the measured photocurrent, limiting spectral rejection while introducing persistent photoconductivity and slower temporal response.

In this work, we demonstrate the monolithic integration of a perovskite filter directly into the photodetector stack to achieve narrowband detection. Specifically, we fabricate a MAPbI_3_ photodetector on top of wider bandgap perovskite layers. Importantly, although the perovskite filter is optically active and absorbs above-bandgap photons, it is electrically decoupled from the photodetector by the insulating layer and therefore does not participate in charge transport or contribute to the photocurrent. This integrated architecture is compared with a more conventional approach, where the filter is placed on the opposite side of the glass substrate. In both cases, narrowband photoresponse is achieved. However, the integrated-filter architecture exhibits a higher EQE, comparable to that of the filterless device in the wavelength range of interest, due to more favorable optical reflection patterns. The monolithically integrated architecture reaches a specific detectivity of 10^13^ Jones at 750 nm and −0.5 V reverse bias. Additionally, the integrated device shows improved stability, maintaining its narrowband response and performance for months under ambient storage.

## Results and Discussion

To investigate the influence of filter integration on the performance of narrowband perovskite PDs, two device architectures were fabricated. Narrowband PDs with integrated-filter (Int-PDs) and external-filter (Ext-PDs), were fabricated as follows. Int-PDs were prepared by monolithically integrating a perovskite filter layer and a perovskite photodetector with the following layout: glass/Cs_*n*_FA_1–*n*_Pb­(I_1–*x*_Br_*x*_)_3_ (900 nm)/Al_2_O_3_ (30 nm)/ITO (80 nm)/TaTm:CS90112 (20 nm)/TaTm (10 nm)/MAPbI_3_ (550 nm)/C_60_ (50 nm)/BCP (7 nm)/Ag (100 nm)/Al_2_O_3_ (30 nm) (Figure S1), where ITO, TaTm, CS90112, and BCP are indium tin oxide, *N*4,*N*4,*N*4′′,*N*4′′-tetra­([1,1′-biphenyl]-4-yl)-[1,1′:4′,1′′-terphenyl]-4,4′′-diamine, 2,2′,2″-(cyclopropane-1,2,3-triylidene)­tris­(2-(cyanotetrafluorophenyl)­acetonitrile) and bathocuproine, respectively. MAPbI_3_ was used as the photoactive layer, while Cs_0.3_FA_0.7_Pb­(I_0.65_Br_0.35_)_3_ or Cs_0.2_FA_0.8_Pb­(I_0.75_Br_0.25_)_3_ were used as filter layers, due to their wider bandgap when compared to MAPbI_3_. The reported layer thicknesses correspond to nominal values controlled during deposition. The spatial uniformity of the deposition platform was independently evaluated using the same vacuum chamber and evaporation sources, showing a maximum thickness deviation of 2.5% across a 4-inch substrate (Figure S2). The different compositions were selected to tune the spectral response of the narrowband PDs. An insulating Al_2_O_3_ layer electrically separates the perovskite filter from the photodetector stack while protecting the former from damage resulting from the deposition of the ITO layer. The TaTm:CS90112 layer was co-evaporated with 13 wt % of dopant, added to increase the electrical conductivity of the hole transport layer. The devices were capped with an additional Al_2_O_3_ layer, used as a protective barrier against moisture and oxygen. For comparison, devices with the filter deposited on the external side of the glass substrate were also fabricated (Ext-PDs, Figure S1b). PDs without an integrated filter were prepared as a reference (Figure S1c). All layers were fabricated by vacuum thermal evaporation, except for ITO, deposited using pulsed laser deposition (PLD), and Al_2_O_3_, deposited by atomic layer deposition (ALD).

First, the optical and structural properties of the perovskite films were characterized. Figure 1a shows the photoluminescence (PL, solid lines) and transmittance (dashed lines) spectra of vapor-deposited Cs_0.3_FA_0.7_Pb­(I_0.65_Br_0.35_)_3_, Cs_0.2_FA_0.8_Pb­(I_0.75_Br_0.25_)_3_, and MAPbI_3_ films. In all cases, the absorption onset coincides with the PL emission maximum, yielding optical bandgaps of 1.77, 1.71, and 1.61 eV, respectively. For simplicity, the two wider-bandgap compositions are hereafter referred to as the 1.8 and 1.7 eV filters, respectively. These values are in good agreement with previous reports for these compositions.^24,25^ Quantitative energy-dispersive spectroscopy (EDS) measurements of the wide-bandgap films yielded Br/(I + Br) ratios of approximately 0.30 and 0.23 for the 1.8 and 1.7 eV filters, respectively, corresponding to approximate AB­(I_0.7_Br_0.3_)_3_ and AB­(I_0.77_Br_0.23_)_3_ halide stoichiometries (Figure S3 and Table S1). The Cs content could not be reliably quantified because of the overlap of its characteristic emission lines with the more intense iodine signal. Accordingly, the perovskite compositions reported throughout the manuscript refer to the nominal compositions used during deposition.

**Figure 1:**
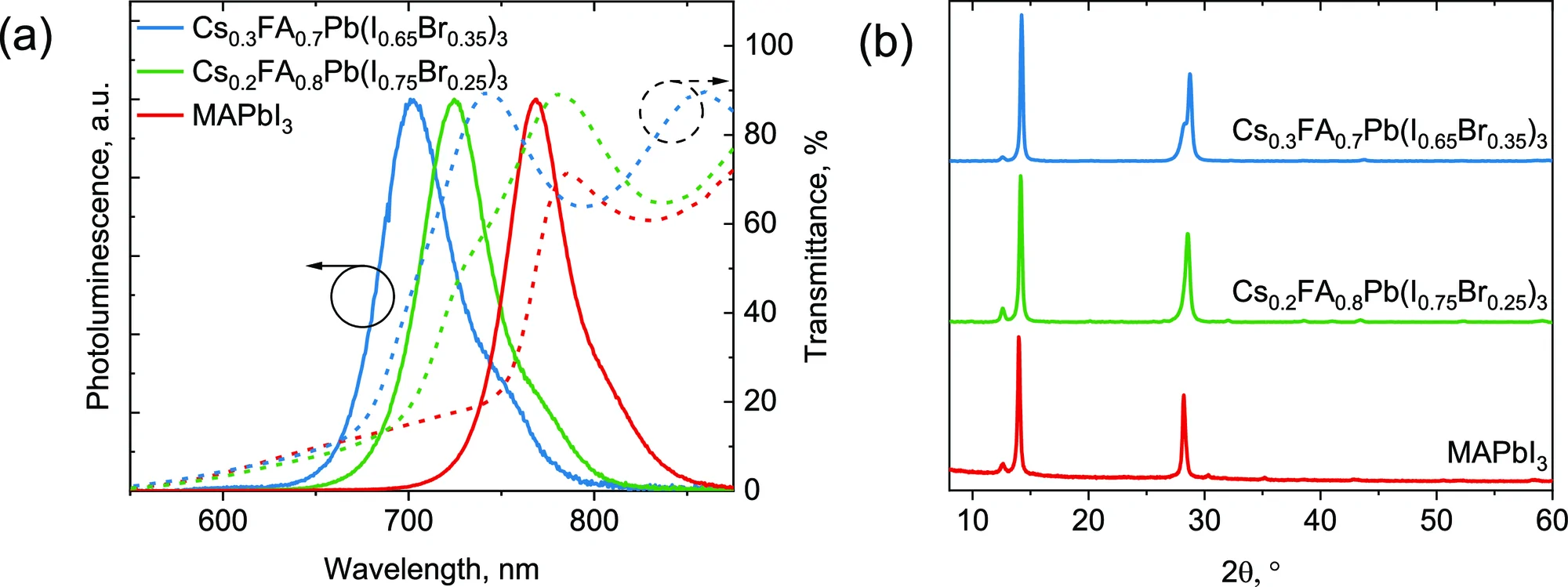
(a) Photoluminescence spectra (left, solid lines), transmittance spectra (right, dashed lines), and (b) X-ray diffraction patterns for vacuum-deposited Cs_0.3_FA_0.7_Pb­(I_0.65_Br_0.35_)_3_, Cs_0.2_FA_0.8_Pb­(I_0.75_Br_0.25_)_3_ and MAPbI_3_ films.

X-ray diffraction (XRD) patterns of the perovskite films were also collected (Figure 1b). All samples show the characteristic peaks of crystalline perovskites in the tetragonal phase, with intense peaks at approximately 14° and 28°, indicating a certain degree of texture. A weak reflection at 12.7° is also observed, corresponding to minor residual PbI_2_.

Next, the complete photodetector stack was studied via cross-section scanning electron microscopy (SEM) and EDS to assess the integration and uniformity of the filters and photoactive layers. Figure 2 shows SEM and EDS analysis of the photodetectors in both configurations, using the 1.7 eV filter layer. The SEM images show, in both cases, compact and continuous layers with large perovskite grains (Figure 2a,b). No voids, delamination or other structural defects are observed throughout the multilayer stack, indicating that the sequential film deposition is compatible with the monolithic device architecture. A more detailed analysis of the surface morphology of these vacuum-deposited wide-bandgap perovskites has been reported in our previous works.^25,26^ The EDS mapping in Figure 2b reveals the Pb, Br, and O signals for the Int-PD: the two well-separated Pb-rich regions correspond to the two distinct perovskite layers (MAPbI_3_ and Cs_0.2_FA_0.8_Pb­(I_0.75_Br_0.25_)_3_), while the Br appears only in the underlying wide bandgap perovskite, indicating no measurable halide interdiffusion between the filter and the photoactive perovskites. Although nanoscale halide migration across the buried interface cannot be excluded, Al_2_O_3_ barrier layers have previously been shown to effectively separate sequentially deposited perovskite films and confine the halide distribution within the individual layers.^27^ The oxygen signal is present in the ITO layer and Al_2_O_3_ layers, as expected, confirming the integrity of the oxide barrier that isolates the filter from the photodetector.

**Figure 2:**
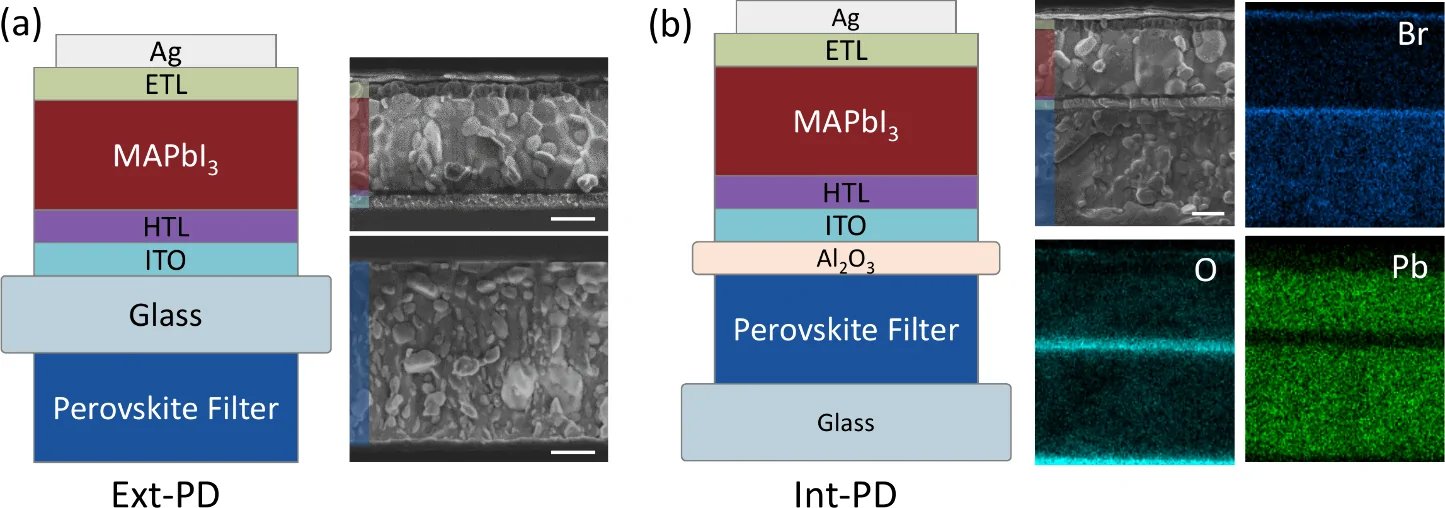
Device layout and cross-sectional SEM images of (a) Ext-PD and (b) Int-PD configurations. On the right side, cross-sectional EDS maps of Int-PD for selected elements (Pb, Br, O). Scales are 250 nm in all microscopy images.

After confirming the quality of the perovskite films and device stacks, we investigated the optoelectronic properties of the photodetectors. Since the goal of this work is to simultaneously maintain a narrowband response with high EQE, we first focused on the spectral EQE of the devices, measured at negative bias (−0.5 V). Both configurations (Int-PD and Ext-PD) were characterized together with a filterless reference device (Figure 3a,b). While the reference device shows a relatively flat spectral response between 60 and 80% across the measured range, both Int-PDs and Ext-PDs exhibit a narrowband response, resulting from the selective absorption of the perovskite filter. For devices incorporating the 1.7 eV filter, the Int-PD configuration reaches a maximum EQE of 78% at 750 nm, comparable to the reference device, with a full width at half maximum (FWHM) of 50 nm (∼0.11 eV). In contrast, Ext-PDs described above exhibit a reduced EQE of 60% at 750 nm and a slightly broader FWHM of 60 nm (∼0.13 eV, Figure 3a). For devices incorporating the 1.8 eV perovskite filter, the photoresponse shifts towards shorter wavelengths and the FWHM increases to 80 nm (∼0.18 eV), as expected from the larger bandgap offset between the filter and the MAPbI_3_ absorber. The Int-PDs again exhibit a higher EQE of 77%, comparable to that of the reference device, with the response peak shifted to approximately 735 nm. In comparison, the Ext-PD reaches only 67% EQE at its maximum response peak. A small absorption tail is observed in the 500–700 nm range for all devices, which could be further suppressed by increasing the filter thickness, as previously demonstrated using external perovskite layers as thick as ∼20 μm.^22^ The spectral rejection ratio (SRR) exceeds 30 dB below 500 nm and, although it decreases toward the narrowband response peak, remains approximately 10 dB at 650 nm, corresponding to a one-order-of-magnitude suppression relative to the peak responsivity (Figure S4). The responsivity spectra of all devices (Figure S5) follow the same trend, with the Int-PDs reaching responsivity comparable to the reference device, whereas the Ext-PDs consistently exhibit lower peak values. Interestingly, these results show that the monolithic integration of the filter results in narrowband photodetection without sacrificing the absolute EQE of the devices. The spectra shown in Figure 3 correspond to representative pixels. Additional EQE spectra were measured for each device configuration at both 0 and −0.5 V, with the individual spectra and the corresponding characteristic parameters reported in Figure S6 and Table S2, respectively. The 1.7 eV Int-PDs measured at −0.5 V exhibit peak EQEs of 76.6–78.5% and FWHM values of 47–51 nm.

**Figure 3:**
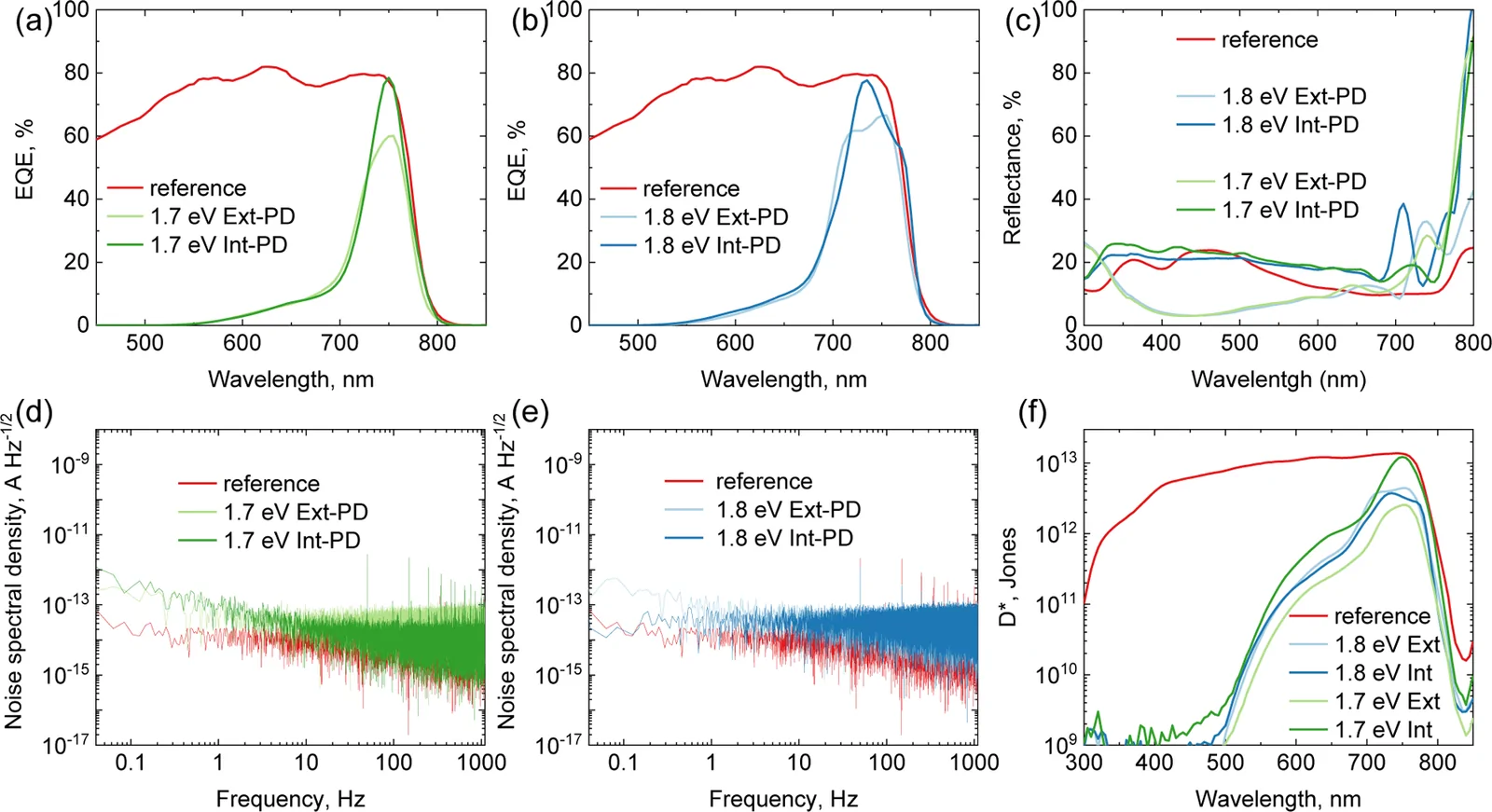
EQE spectra of devices with (a) 1.7 eV and (b) 1.8 eV perovskite filters, in both studied configurations. The EQE spectrum of a reference filterless device is also shown for comparison. (c) Reflectance spectra of all devices studied in this work. Noise current spectral density of devices with (d) 1.7 eV and (e) 1.8 eV perovskites, compared with the reference device. (f) Specific detectivity of all devices studied in this work.

To understand the origin of the different EQE values between the two configurations, we measured the reflectance of the full stack using an integrating sphere. As shown in Figure 3c, the Int-PD configuration exhibits a reflectance profile similar to that of the reference device, whereas the Ext-PD displays a markedly different spectral shape. The most relevant difference occurs near the EQE maximum (ca. 750 nm), where the Int-PD shows a reflectance minimum, while the Ext-PD exhibits a maximum. The higher reflectance of the Ext-PD around the narrowband response maximum is therefore consistent with increased optical losses and its lower EQE. Additional reflectance measurements of the isolated filters from opposite illumination directions show that the filter position strongly affects the reflectance spectrum (Figure S7), further supporting an optical origin for the higher EQE of the Int-PD.

We measured the noise current (*i*_noise_) spectral density of representative narrowband photodetectors at −0.5 V up to 1 kHz and compared the results with those of the reference device (Figure 3d,e). All devices exhibit similar noise characteristics: a frequency-dependent 1/*f* component dominating below approximately 1–10 Hz, followed by a frequency-independent white-noise region arising from Johnson and shot noise. In this frequency range, all devices exhibit comparable noise current spectral densities (between 1 and 4 × 10^–14^ A/√Hz ), indicating that integrating the filter does not introduce additional electrical noise.

Hence, we calculated the specific detectivity (D*), one of the key figures of merit for photodetectors (Figure 3f). To compare the different devices, the specific detectivity values were extracted at 750 nm and 182 Hz. For the 1.8 eV filter, D* values of 4 × 10^12^ Jones and 3 × 10^12^ Jones were obtained for the Ext-PD and Int-PD, respectively. Using the 1.7 eV perovskite filter, D* values are 2.5 × 10^12^ Jones for the Ext-PD, and up to 10^13^ Jones for the Int-PD. Since all devices exhibit nearly identical noise levels, these differences mainly reflect the higher responsivity of the Int-PD architecture. The detectivity of the best-performing Int-PDs matches that of the filterless reference photodiode (1 × 10^13^ Jones), despite its much narrower spectral response. Consequently, these values compare favorably with those reported for perovskite narrowband photodetectors,^28^ highlighting that monolithic filter integration enables spectral selectivity without compromising the detector performance. It is worth noting that these are maximum D* values, as the detectivity rapidly decreases toward both shorter and longer wavelengths away from the response peak.

The dynamic response of the narrowband photodetectors was also investigated by transient and frequency-dependent measurements (Figure S8). The devices exhibit fast and comparable rise and fall times (3–4 μs), consistent with a response predominantly limited by the device capacitance. Small differences between devices may arise from variations in the effective excitation density, as the 750 nm LED used here has a finite spectral width and therefore a different overlap with the narrowband response of each detector. Importantly, the transient response is consistent with the independently measured −3 dB bandwidth (>130 kHz), in agreement with the expected relationship between the 10–90% step response and bandwidth for a first-order system.

To quantify the linearity of the photodetectors, the linear dynamic range (LDR) was estimated from measurements using a white LED combined with a 750 nm bandpass filter as the illumination source. The incident light was modulated at 182 Hz to avoid contributions from 1/*f* noise as well as power-line noise and its harmonics. The light intensity was attenuated using a set of neutral density filters, and the device response was measured in the frequency domain at −0.5 V for the devices using the 1.8 eV perovskite filter (Figure 4a). Both Int-PDs and Ext-PDs exhibit a linear response over approximately five orders of magnitude, corresponding to an LDR of 100 dB. Power-law fitting (*I* ∝ *P* ^α^), yields exponents 0.996 and 0.998 for the Ext-PD and Int-PD, respectively, confirming an ideal linear response. It is important to note that the measurable intensity range was limited by the available light source, hence, the reported value of the LDR should be taken as a lower bound. Figure 4b shows representative frequency-domain spectra of the Int-PD under different illumination intensities. The photocurrent is detected as a distinct peak at the modulation frequency (182 Hz), which progressively emerges above the noise floor with increasing illumination intensity. This measurement approach enables accurate determination of the minimum detectable signal while avoiding contributions from low-frequency and power-line noise.

**Figure 4:**
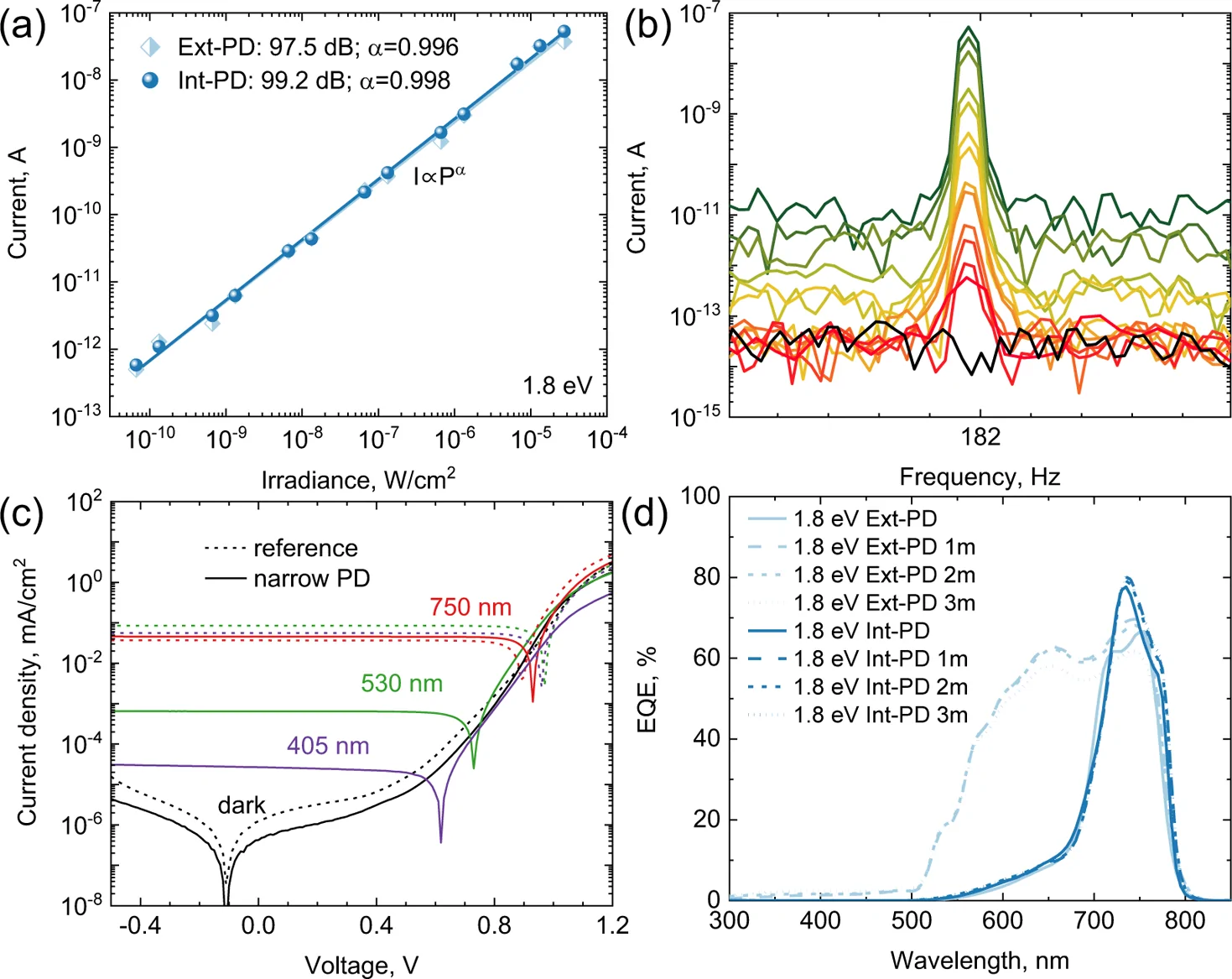
(a) AC-mode linear dynamic range at 750 nm illumination for devices with 1.8 eV perovskite filter, extracted from the (b) photocurrent response of 1.8 eV Int-PD device to a 182 Hz modulated light source plotted versus frequency. (c) *I*–*V* curves under illumination of different light wavelengths (405, 530, 750 nm) and in the dark condition of reference and narrow 1.8 eV Int-PD. (d) Comparison of EQE spectra for 1.8 eV Ext-PD and Int-PD as-prepared and after ambient storage for 1–3 months.

To further highlight the spectral selectivity of the narrowband photodetectors, we performed current–voltage (*I*–*V*) measurements in the dark and under illumination at different wavelengths, namely, 405, 530, and 750 nm (Figure 4c) on the 1.8 eV Int-PD and the reference filterless device. For all wavelengths, the incident optical power was kept at approximately 5 × 10^–5^ W. At −0.5 V, the reference and Int-PD exhibit dark currents of 1 × 10^–9^ and 4 × 10^–10^A, respectively, while under 750 nm illumination the corresponding photocurrents are 3 × 10^–6^ and 4 × 10^–6^A. This comparable electrical response confirms that the integrated filter is electrically isolated from the photodetector stack and does not adversely affect its operation. A slight shift of the dark *I*–*V* characteristics near 0 V is also observed. This behaviour is commonly found in low-dark-current perovskite photodiodes, where slow transient effects associated with the mixed ionic–electronic nature of the perovskite become more apparent.^29^ In contrast, when illuminated with 530 and 405 nm light, the reference device shows photocurrent comparable to that measured under 750 nm illumination, whereas the narrowband device displays a significantly suppressed response, approximately two orders of magnitude lower at 530 nm and nearly three orders of magnitude lower at 405 nm, approaching the dark current level. These results confirm that the integrated filter efficiently suppresses the response to above-bandgap photons while preserving the near-band-edge photoresponse.

Finally, we studied the shelf stability of the photodetectors. The photodiodes were encapsulated using UV-curable glue and a cover glass before storage under ambient conditions. In the Int-PD architecture, the perovskite filter is contained within this glass-to-glass encapsulated stack, whereas in the Ext-PD configuration the filter remains external to the encapsulated photodiode. A small decrease in the forward diffusion current is observed after approximately 10 months of ambient shelf storage, particularly for the Int-PD. However, the reverse-bias dark current is largely maintained for both device architectures (Figure S9). Photographs of the aged devices are shown in Figure S10, showing a clear color change for the Ext-PD after 1 month in ambient atmosphere. The EQE spectra measured after shelf storage confirm this difference in stability (Figure 4d). The Int-PD has an essentially unchanged spectral response after over three months of storage. In contrast, after one month, the Ext-PD has already largely regained its broadband response in the 500–700 nm range. This indicates degradation of the externally coated perovskite filter, whereas integrating the filter within the device stack effectively preservesits functionality over time.

## Conclusion

In conclusion, we demonstrate a monolithically integrated narrowband detector in which a perovskite photodiode is fabricated directly on top of an electrically passive perovskite filter. We show that by adjusting the composition of the perovskite filter layer, the narrowband response can be readily tuned. Using this method, we achieved a narrow spectral response with a 50 nm FWHM centred at 750 nm, while maintaining an EQE of up to 78% and a specific detectivity of 10^13^ Jones. These figures of merit are comparable to those of the filterless reference device and superior to those obtained using the conventional external-filter architecture, demonstrating that narrowband operation can be achieved without compromising the performance of the underlying photodetector. Moreover, devices with the integrated filter show enhanced shelf stability under ambient conditions, preserving their narrowband response and photodetector performance after storage. Overall, this work demonstrates that monolithic integration of an electrically passive perovskite filter is an effective strategy for realizing high-performance, stable narrowband photodetectors.

## Materials and Methods

### Materials

*N*4,*N*4,*N*4″,*N*4″-Tetra­([1,1′-biphenyl]–4-yl)-[1,1′:4′,1″-terphenyl]-4,4′′-diamine (TaTm), CsI and PbBr_2_ were purchased from Tokyo Chemical Industry. Fullerene (C_60_) was purchased from M. Braun GmbH. Methylammonium iodide (MAI), bathocuproine (BCP) and 2,2′,2″-(cyclopropane-1,2,3-triylidene)­tris­(2-(*p*-cyanotetrafluorophenyl)­acetonitrile) (CS90112) were purchased from Luminescence Technology Corp. PbI_2_ (99.999%, beads) was purchased from Sigma-Aldrich. Formamidinium iodide (FAI) was obtained from Greatcell Solar Materials. All materials were used as received.

### Device Fabrication

The glass substrates were cleaned subsequently with soap, water, and isopropanol in an ultrasonic bath, followed by 20 min of UV–ozone treatment. The substrates were then transferred to a vacuum chamber integrated within a nitrogen-filled glovebox and evacuated to a pressure of 10^–6^ mbar for perovskites and organic charge-transport layers deposition. The perovskite filter layer, perovskite active layer, and transport layers were each deposited in separate vacuum chambers.

The perovskite filter layers were evaporated in a dedicated vacuum chamber, equipped with four evaporation sources (MBraun Inertgas-systeme GmbH) with independent temperature controllers and shutters. All sources have a dedicated quartz crystal microbalance (QCM) sensor above, which was individually calibrated for the corresponding material. The materials are loaded in alumina crucibles. The mixed halide Pb­(I_1–*x*_Br_*x*_)_2_ precursor was prepared by mixing in an alumina crucible the calculated amounts of PbI_2_ and PbBr_2_, and by heating them at 380 °C for 5 min, when complete melting of the mixture is achieved. During the Cs_0.2_FA_0.8_Pb­(I_0.75_Br_0.25_)_3_ perovskite deposition, the deposition rates of FAI, CsI and Pb­(I_1–*x*_Br_*x*_)_2_ were kept constant at 0.45 Å s^–1^, 0.1 Å s^–1^ and 0.45 Å s^–1^. The pressure of the chamber was maintained at 8 × 10^–6^ mbar. The temperature of the substrates was at −20 °C. Typical sublimation temperatures for the precursors were 150 °C for FAI, 260 °C for the Pb­(I_1–*x*_Br_*x*_)_2_ mixture and 410 °C for the CsI. To tune the bandgap, we changed the amount of bromide in the alloy (PbBr_2_:PbI_2_): for Cs_0.3_FA_0.7_Pb­(I_0.65_Br_0.35_)_3_ (1:6) and for Cs_0.2_FA_0.8_Pb­(I_0.75_Br_0.25_)_3_ (1:8). The rate of CsI was also changed: 0.15 Å s^–1^ for Cs_0.3_FA_0.7_Pb­(I_0.65_Br_0.35_)_3_ and 0.1 Å s^–1^ for Cs_0.2_FA_0.8_Pb­(I_0.75_Br_0.25_)_3_.

After deposition of the filter perovskites, the samples were coated with 30 nm of Al_2_O_3_ deposited by atomic layer deposition (Arradiance GEMStar XT thermal ALD), using a previously published protocol.^30^ The ITO layer was deposited by pulsed laser deposition (PLD) using a Solmates large-area 200 mm PLD system at a chamber pressure of 0.033 mbar, as described in a previous work.^31^

The doped TaTm:CS90112 hole-transport layer was co-evaporated at deposition rates of 0.8 Å/s (TaTm) and 0.12 Å/s (CS90112), corresponding to a CS90112 ratio of ∼13%. The intrinsic transport layers (TaTm, C_60_, BCP) were deposited at a rate of 0.3 Å/s.

For the MAPbI_3_ deposition, MAI and PbI_2_ were co-evaporated at rates of 0.22 Å/s and 0.67 Å/s, respectively. Two evaporation sources (one each for PbI_2_ and MAI) and three quartz crystal microbalance (QCM) sensors were used: two QCMs monitored the individual source rates and a third QCM placed near the substrate holder monitored the total deposition rate. During co-evaporation, the chamber pressure was maintained at 5 × 10^–6^ mbar.^32^ Ag electrodes were thermally evaporated in a dedicated chamber using alumina-coated molybdenum boats with applied currents ranging from 20 to 30 A. For the Ag contacts, a shadow mask defined a pixel area of 0.0825 cm^2^. The whole device was encapsulated with 30 nm of Al_2_O_3_.

### Characterization of Materials and Devices

The photoluminescence spectra were measured with an Avantes Avaspec2048 spectrometer, and films were illuminated with a diode laser of Integrated Optics, emitting at 405 nm. Transmittance measurements were collected using a PerkinElmer Lambda 950 UV–vis- NIR spectrometer. This system is coupled to an integrating sphere.

The XRD patterns were collected in Bragg–Brentano geometry on an Empyrean PANalytical powder diffractometer with a copper anode operated at 45 kV and 40 mA.

The SEM images were taken with a Field Emission Scanning Electron Microscope (HR-FESEM), ZEISS GeminiSEM 500 model, with a secondary Electron In-Lens detector using an accelerating voltage of 0.7–1 kV. The EDS images were obtained using the same equipment provided with an X-ray Dispersive Energy Detector (Oxford Instruments).

EQE and device reflectance measurements were performed on a QE-R system from Enlitech. The system was calibrated using a calibrated silicon reference cell. The EQE was measured as a function of wavelength from 300 to 850 nm in 5 nm steps. Device reflectance was measured with the same system with an integrating sphere.

The *I*–*V* characteristics of the photodiodes were measured using a Keithley 2450 SourceMeter over a voltage range of −0.5 to 1.2 V with 0.01 V steps. As light sources, 405 and 530 nm LEDs (M405L4, M530L4, ThorLabs) were used directly, while white LEDs (MBB1L3, ThorLabs) were used in combination with a 750 nm narrow-band filter to obtain narrow 750 nm illumination. All LEDs were driven by a Thorlabs DC2200 LED driver. Light intensities were calibrated prior to each measurement using a certified silicon reference photodiode.

Noise current measurements were carried out inside a KeyFactor Faraday cage. The signal from the DUT was connected through a FEMTO low-noise cable to a FEMTO DLPCA-200 low-noise transimpedance amplifier, which provides selectable gain and adjustable bias voltage. The amplified signal was then recorded using a Rohde & Schwarz RTA4004 oscilloscope, with the resolution bandwidth set to 1 Hz.

The same measurement setup was employed for determining the linear dynamic range, except that the DUT was illuminated using white LEDs combined with a 750 nm narrow-band filter and driven at 182 Hz by an external driver (Thorlabs, DC2200). The optical power was adjusted using a series of neutral-density filters. For the LDR measurements, absolute current rather than current density was used, since a single illuminated area cannot be defined consistently over the full irradiance range: at high irradiance a shadow mask would be required to restrict illumination to the pixel active area, whereas at the lowest irradiances the measured current approaches the dark-current regime.

Time-response and cut-off frequency measurements were performed with a 750 nm LED (≈4 × 10^–4^ W cm^–2^) modulated by an Agilent 33521A function/arbitrary waveform generator; the photocurrent was recorded on a Rohde & Schwarz RTA4004 oscilloscope.

## Supplementary Material



## Notes

Device layouts, spatial uniformity of perovskite films, EDS characterization analysis, spectral rejection ratio and responsivity spectra, reflectance measurements, time response, device photographs, and definitions of photodetector figures of merit (PDF)
