# Supplementary material for: Monolithically Integrated Perovskites for High-Performance Vapor-Deposited Narrowband Photodetectors
Source: ACS Photonics. 2026 Sep 28;13(19):5868–75. doi: 10.1021/acsphotonics.6c01728 (PMC13660377; doi:10.1021/acsphotonics.6c01728)
Supplement: Supplementary file 1 [file ph6c01728_si_001.pdf]

Supporting Information

Monolithically integrated perovskites for high-performance vapor-deposited narrowband photodetectors

Serhii Derenko, Lidón Gil–Escrig, Joost W. C. Reinders, Cristina Roldán–Carmona, Daniel Tordera, Michele Sessolo*

Instituto de Ciencia Molecular, Universidad de Valencia, C/ Catedrático J. Beltrán 2, 46980 Paterna, Spain

Contents

Figure S1. Schematic layouts of Int-PD, Ext-PD, and reference device configurations — **S2**

Figure S2. Spatial uniformity of vacuum-deposited perovskite films — **S3**

Figure S3. EDS spectra of the 1.7 and 1.8 eV perovskite filter films — **S3**

Table S1. Br/(I+Br) atomic ratios from quantitative EDS analysis — **S4**

Figure S4. Spectral rejection ratio of Int-PD and Ext-PD devices — **S4**

Figure S5. Spectral responsivity of the devices — **S4**

Figure S6. Pixel-to-pixel EQE reproducibility of Int-PD and Ext-PD devices — **S5**

Table S2. EQEmax, λmax, and FWHM values extracted from Figure S6 — **S6**

Figure S7. Reflectance spectra of the perovskite filters from opposite illumination directions — **S6**

Figure S8. Temporal and frequency response of the narrowband photodetectors — **S7**

Figure S9. Dark current–voltage characteristics before and after approximately 10 months of ambient storage — **S7**

Figure S10. Photographs of aged Ext-PD and Int-PD devices — **S8**

Definitions. Photodetector figures of merit and calculation formulas — **S9**

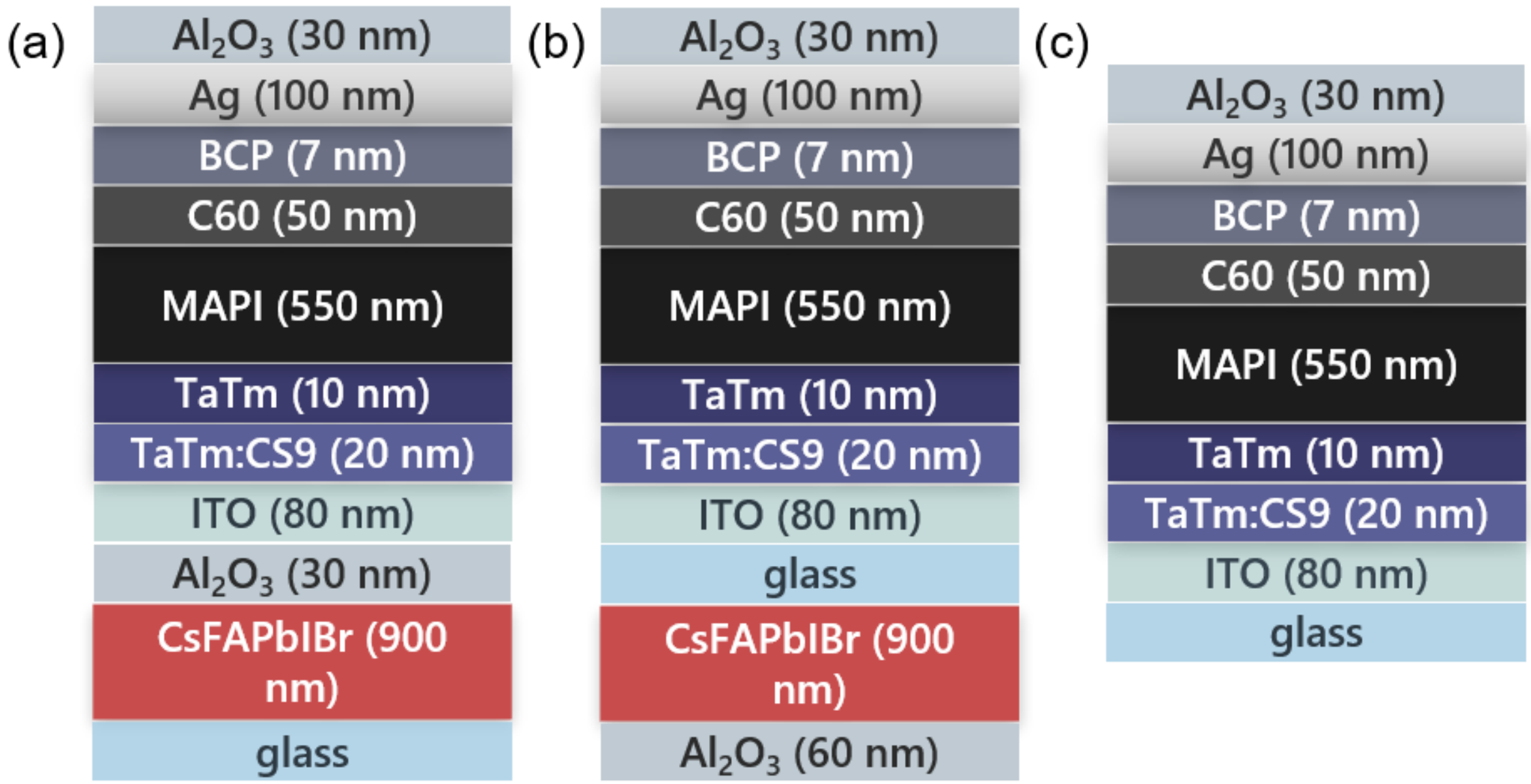

Figure S1. Schematic layout of (a) Int-PD configuration, (b) Ext-PD configuration, (c) reference vacuum-processed devices used in this work

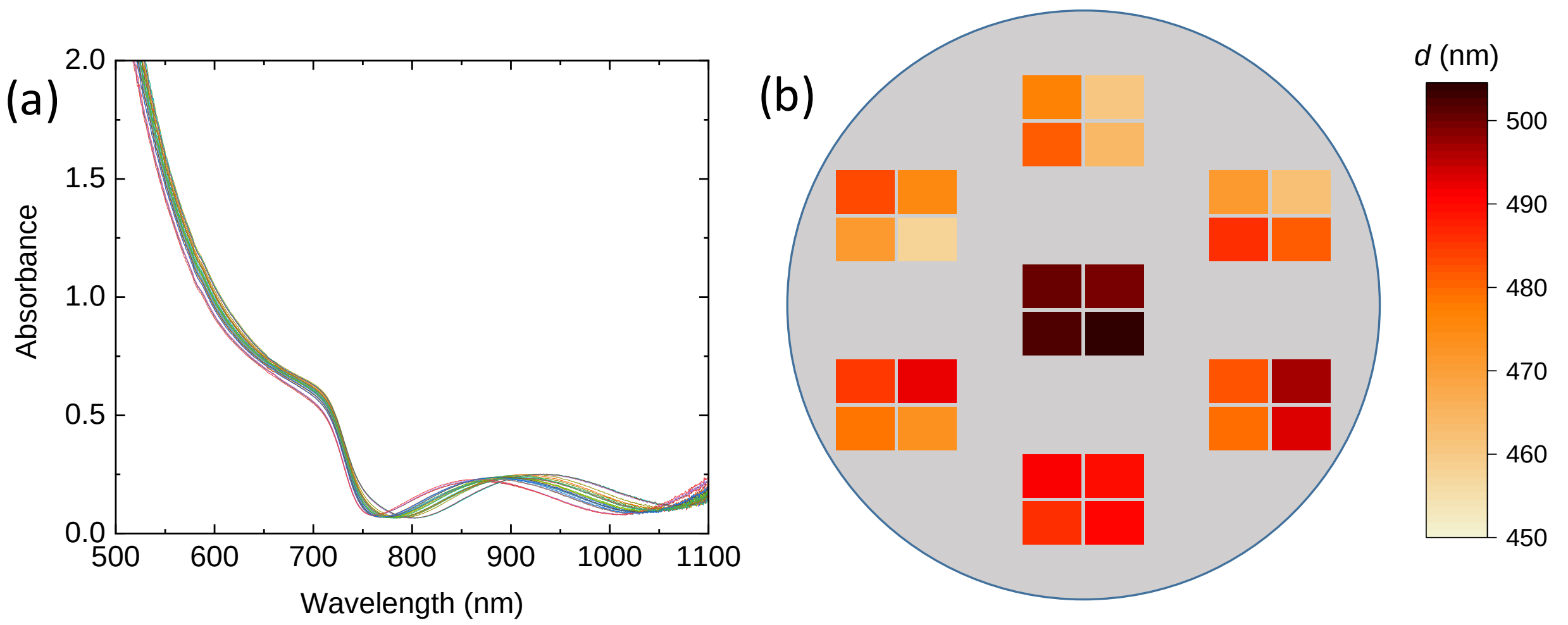

Figure S2. Spatial uniformity of a vacuum-deposited perovskite film prepared using the same deposition chamber and evaporation sources employed in this work. (a) Optical absorption spectra measured at 28 positions across a 4-inch substrate. (b) Thickness map measured at the same positions. The average film thickness is 480 nm, with a maximum deviation of 2.5% across the substrate.

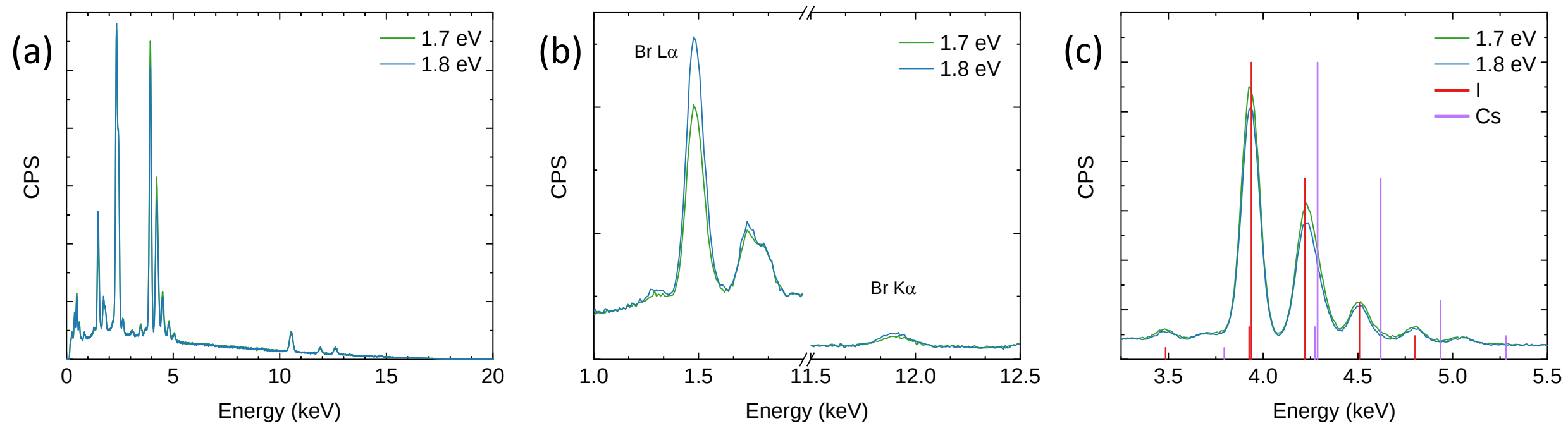

Figure S3. Representative EDS spectra of the 1.7 and 1.8 eV perovskite filter films. (a) Full spectra and (b) enlarged Br spectral region, showing the higher Br signal of the 1.8 eV film. (c) Enlarged region containing the characteristic I and Cs emission lines; vertical markers indicate the expected positions of I (red) and Cs (purple) transitions. The strong spectral overlap between the I and Cs signals, together with the much higher iodine content, prevents reliable quantitative determination of the Cs concentration.

Table S1. Br/(I+Br) atomic ratios obtained by quantitative EDS analysis at three different regions of each wide-bandgap perovskite film.

Filter	Region 1	Region 2	Region 3	Average x in $AB(I_{1-x}Br_x)$
1.8 eV	0.302	0.297	0.298	~ 0.30
1.7 eV	0.227	0.228	0.230	~ 0.23

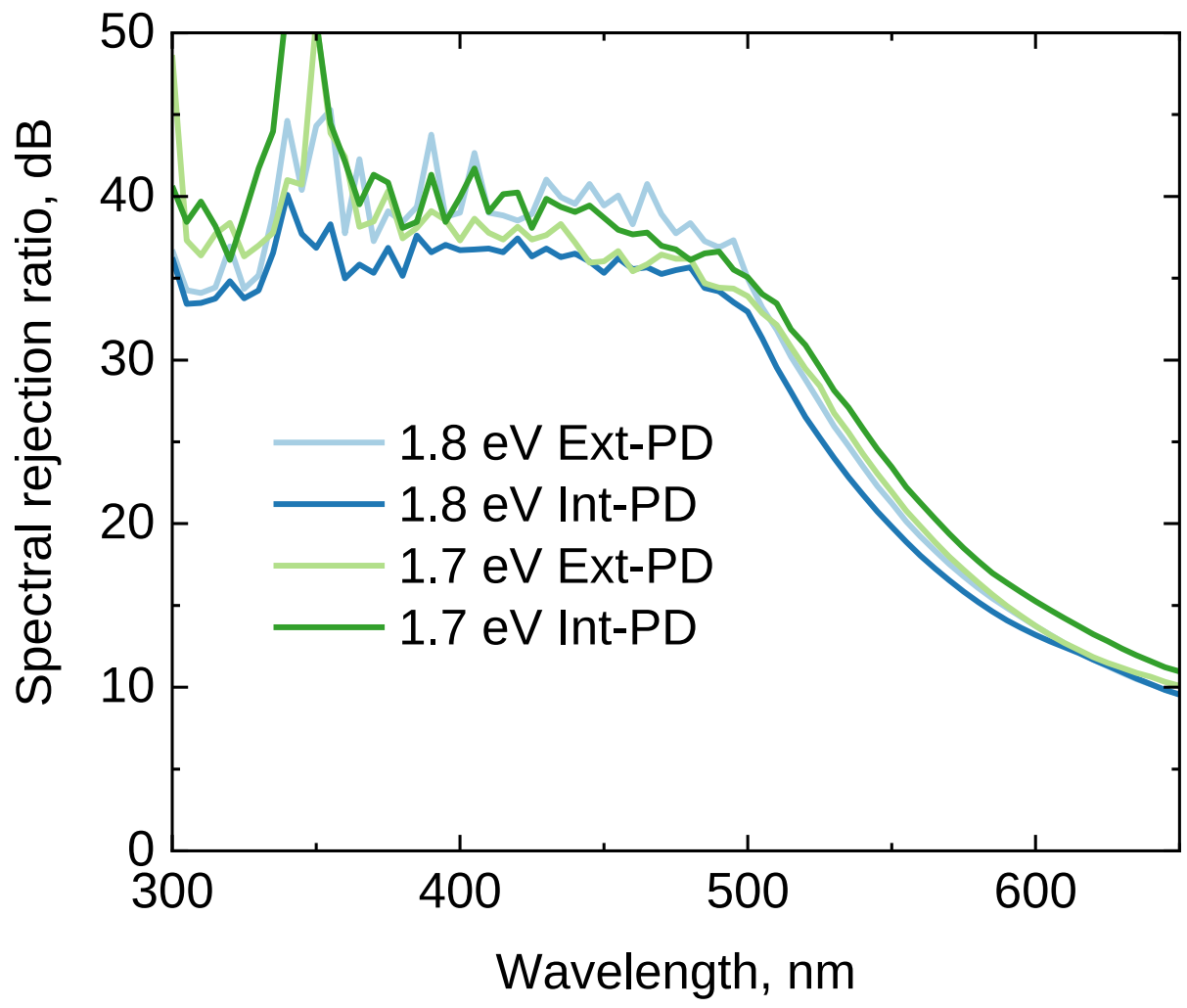

Figure S4. Spectral rejection ratio (SRR) of the 1.7 and 1.8 eV Ext-PDs and Int-PDs, calculated as $10\ \log_{10}[R_{max}/R(\lambda)]$. The SRR exceeds 30 dB below 500 nm and decreases progressively toward the narrowband response peak, remaining around 10 dB at 650 nm.

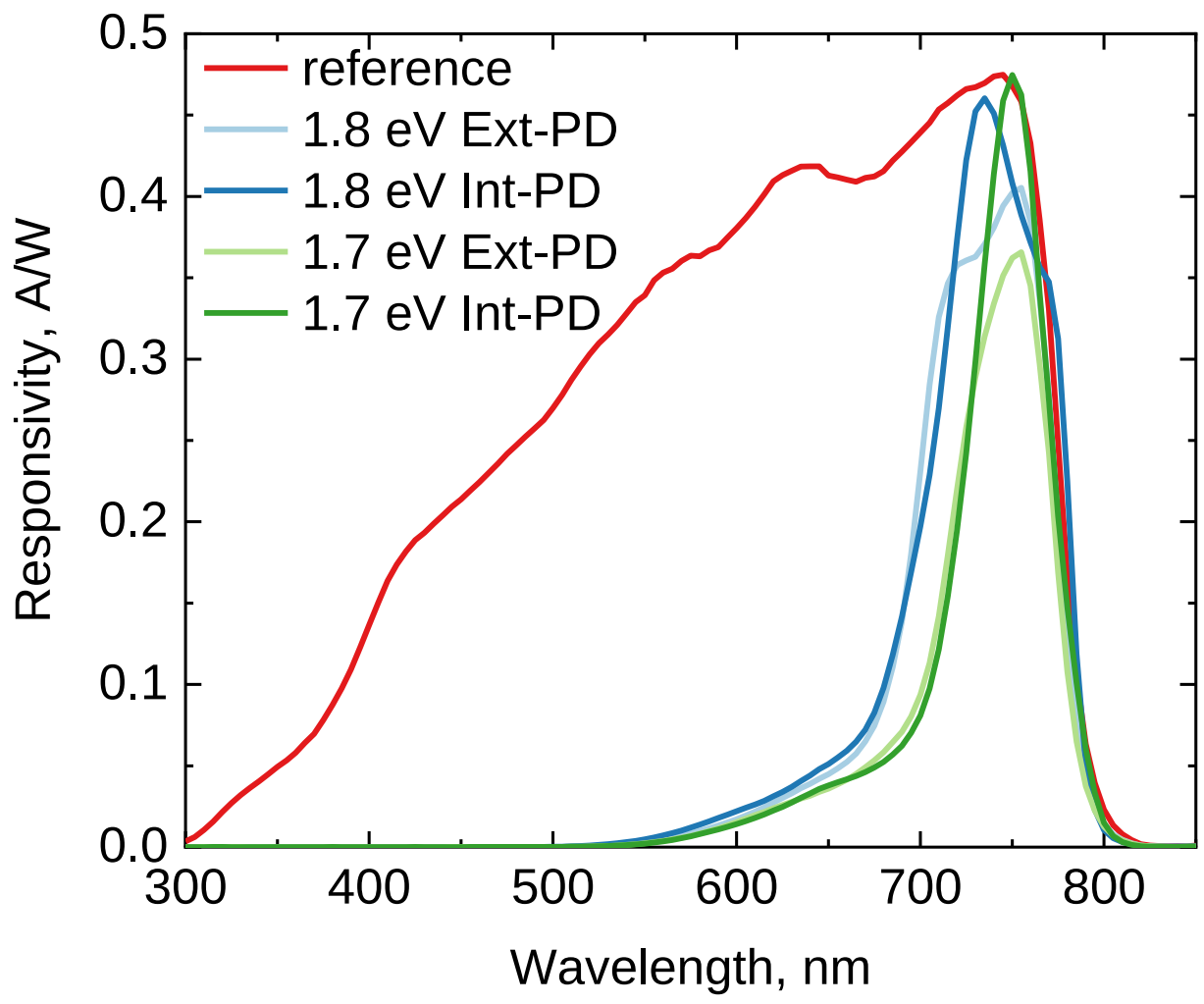

Figure S5. Spectral responsivity of devices used in this work

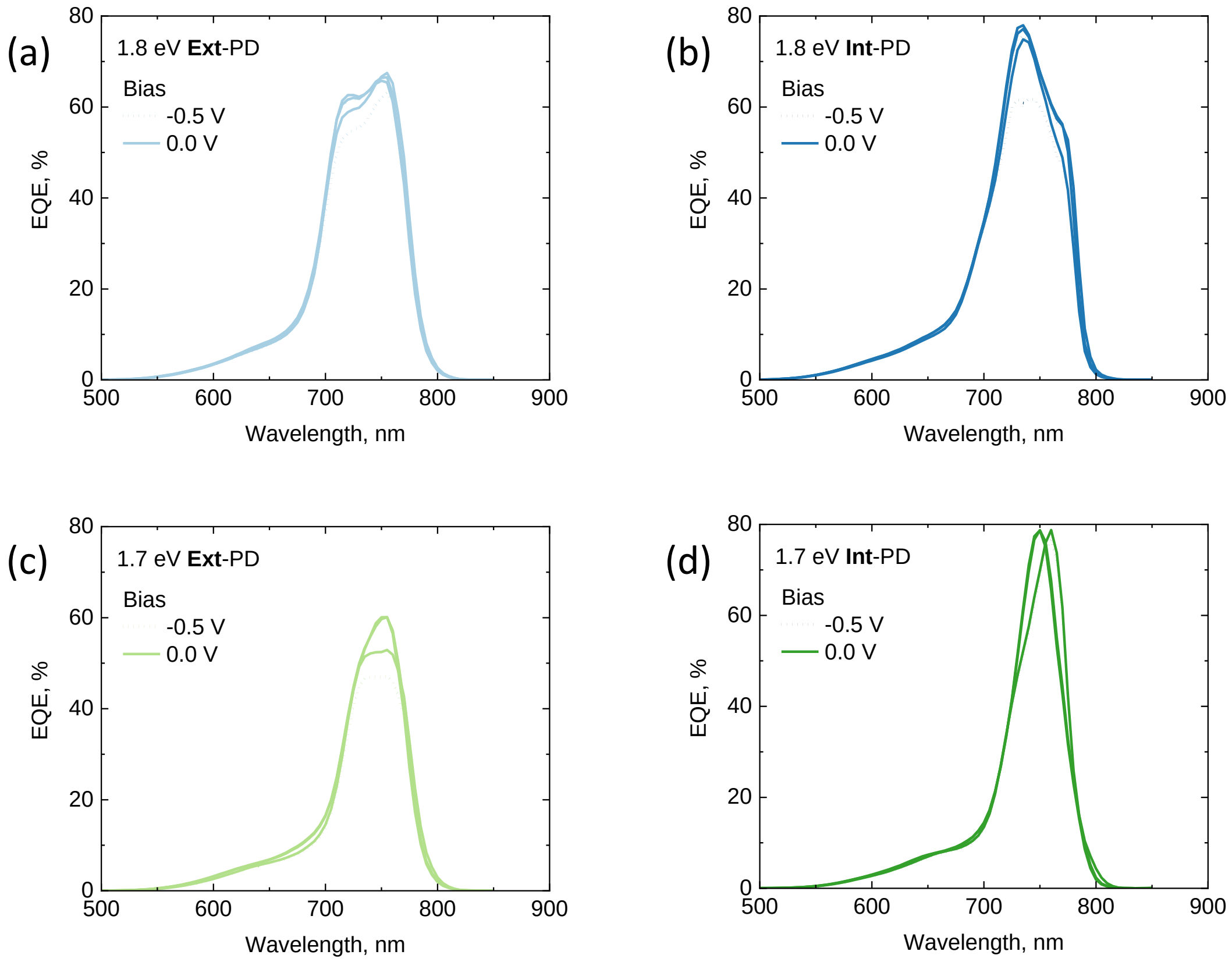

Figure S6. EQE spectra of three pixels from different devices fabricated within the same deposition run for each Ext-PD and Int-PD configuration incorporating the 1.8 and 1.7 eV perovskite filters, measured at 0 and −0.5 V.

Table S2. Peak external quantum efficiency (EQE_{max}), wavelength of maximum response (λ_{max}), and full width at half maximum (FWHM) extracted from the EQE spectra shown in Figure S6. EQE spectra were acquired in 5 nm wavelength steps.

Bias (V)	Type	EQE_{max} (%)	λ_{max} (nm)	FWHM (nm)	Type	EQE_{max} (%)	λ_{max} (nm)	FWHM (nm)
-0.5	1.8 Ext-PD	65.7	750	79	1.8 Int-PD	61.7	730	82
		63.2	755	81		75.7	735	78
		66.6	755	78		77.7	735	76
0.0		65.8	750	77		74.5	735	73
		67.5	755	79		77.1	735	77
		66.5	755	78		78	735	76
-0.5	1.7 Ext-PD	47	745	65	1.7 Int-PD	76.6	760	51
		60.1	755	60		78.5	750	47
		60.3	755	61		78.5	750	48
0.0		52.9	755	65		78.8	760	52
		60.1	755	60		78.7	750	49
		60.1	755	60		78.7	750	48

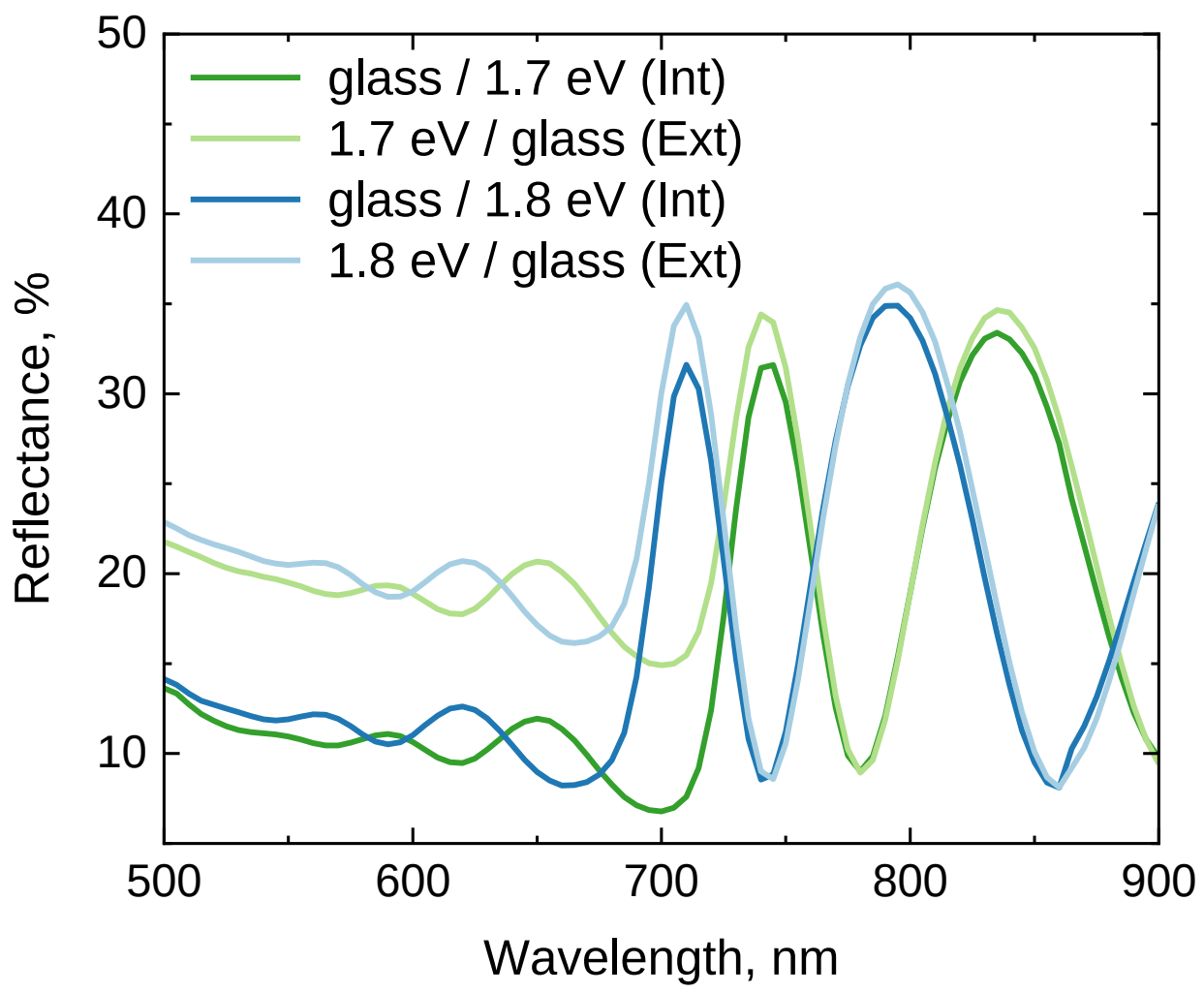

Figure S7. Reflectance spectra of the 1.7 and 1.8 eV perovskite filters deposited on glass, measured with illumination incident either through the glass (solid lines) or directly onto the perovskite layer (dashed lines).

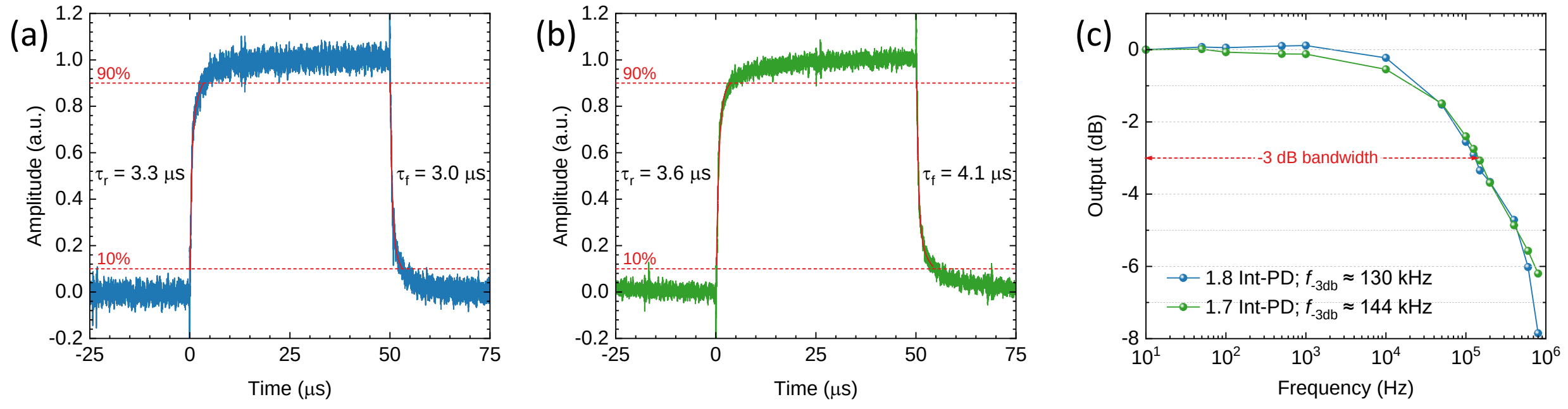

Figure S8. Temporal and frequency response of the narrowband photodetectors. (a, b) Transient photocurrent response under 750 nm LED illumination at an irradiance of $\approx 4{\cdot}10^{-4}$ W cm^{-2}. Red curves show fits to the rising and falling edges, used to determine the 10% and 90% signal crossing points and extract the corresponding rise and fall times. (c) Normalized frequency-dependent photoresponse of the devices, with the −3 dB level indicated for determination of the bandwidth. The transient response and frequency-domain measurements provide consistent estimates of the device response speed.

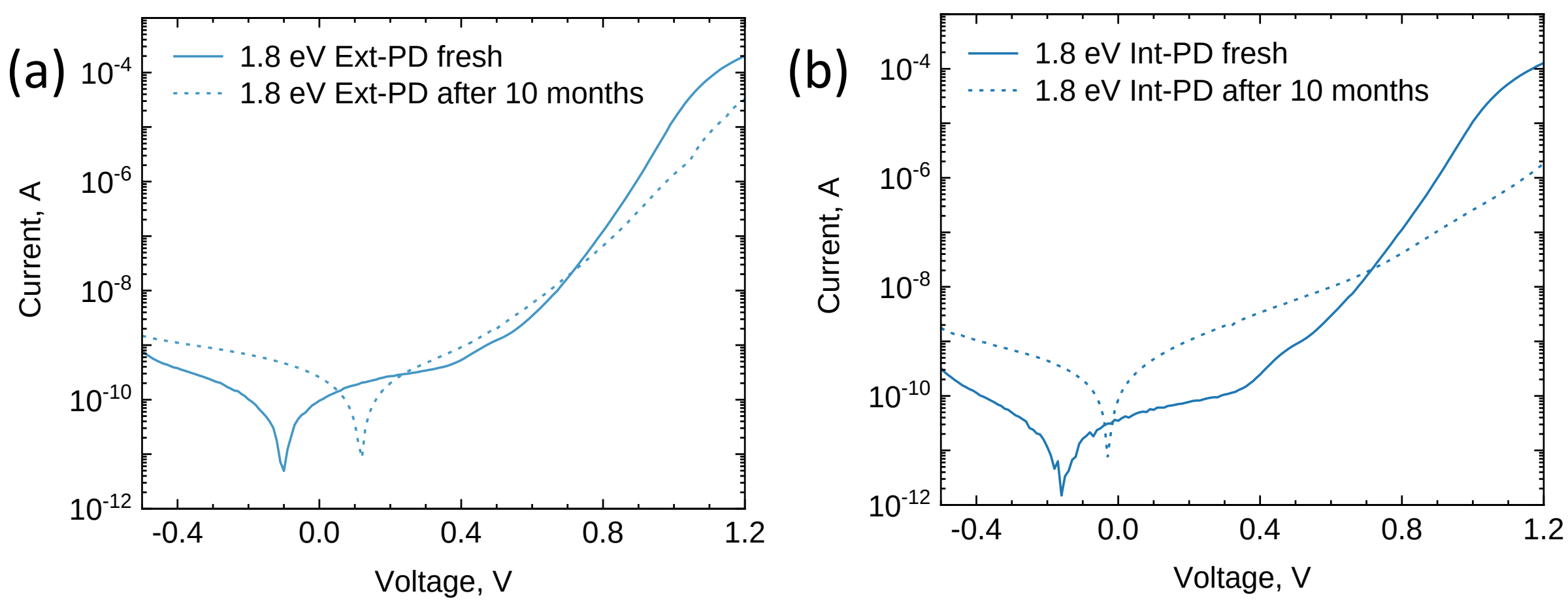

Figure S9. Dark current–voltage characteristics of the 1.8 eV (a) Ext-PD and (b) Int-PD measured as prepared and after approximately 10 months of ambient shelf storage.

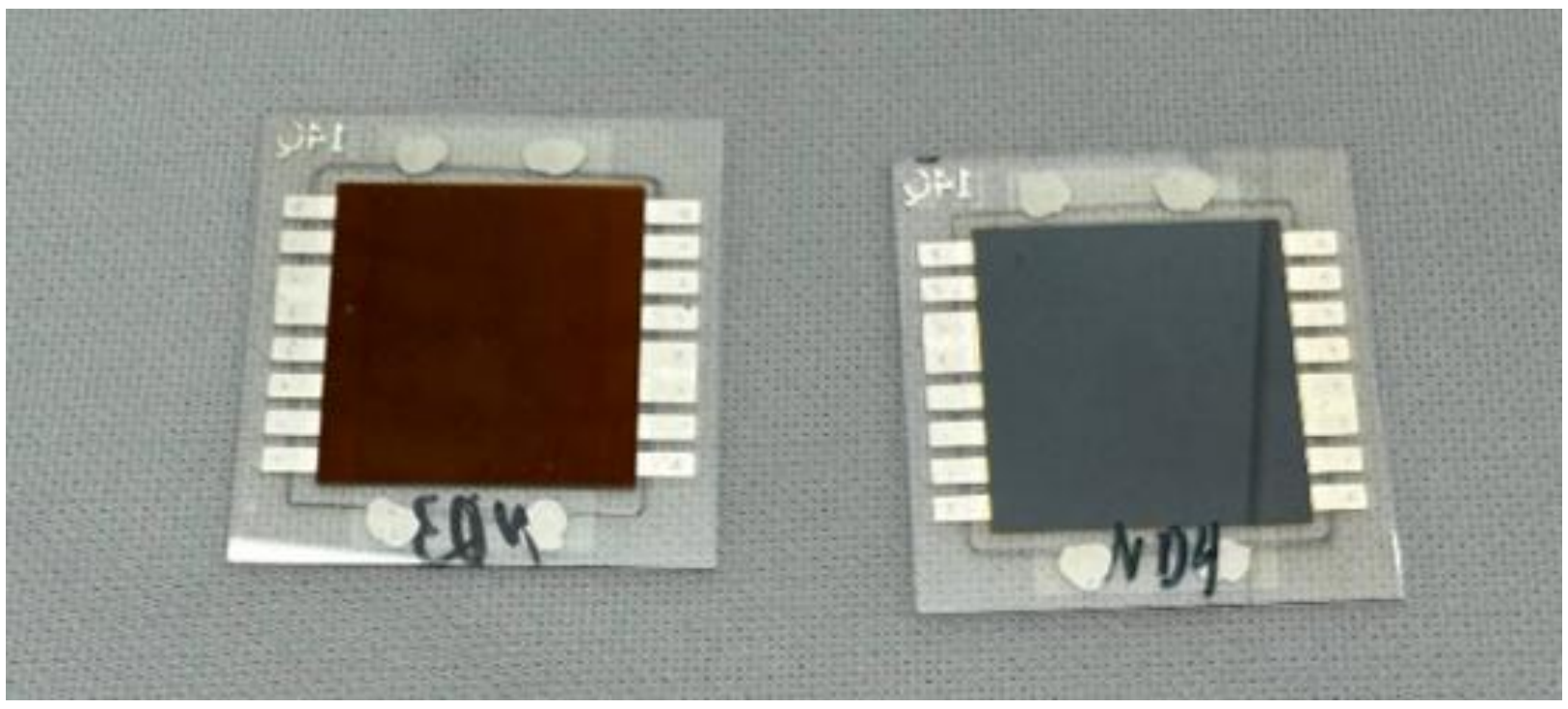

Figure S10. Photo of two devices with $Cs_{0.3}FA_{0.7}Pb(I_{0.65}Br_{0.35})_3$ perovskite after one month of shelf storage. Left: 1.8 eV Ext-PD; right: 1.8 eV Int-PD

Definitions

The responsivity is the photocurrent I_{ph} generated by a detector in response to an optical power P_{in}

$$R = \frac{I_{ph}}{P_{in}}$$

The external quantum efficiency (EQE) of a photodetector is the ratio of the number of charge carriers collected by the device to the number of incident photons shining on it at a specific energy. It is conveniently calculated from R and E_{ph}

$$EQE = R \cdot E_{ph}$$

D^* represents the detector's signal-to-noise ratio normalised by its active area and noise bandwidth, and is given by the following equation:

$$D^* = \frac{R\sqrt{A}}{i_{noise}}$$

where R is the responsivity, A is the device active area, i_{noise} is the noise-current spectral density.

To quantify the linearity of the photodetectors, the linear dynamic range (LDR) was estimated using the following equation:

$$LDR = 20 log \frac{I_{max}}{I_{min}}$$

where I_{max} and I_{min} correspond to the maximum and minimum photocurrent values measured as a function of the irradiance, within the linear operating regime.
